# Determination of S-Adenosylmethionine and S-Adenosylhomocysteine by LC–MS/MS and evaluation of their stability in mice tissues

**DOI:** 10.1016/j.jchromb.2009.05.039

**Published:** 2009-07-15

**Authors:** Jakub Krijt, Alena Dutá, Viktor Kožich

**Affiliations:** Institute of Inherited Metabolic Disorders, Charles University in Prague — 1st Faculty of Medicine, Prague, Czech Republic

**Keywords:** S-Adenosylmethionine, S-Adenosylhomocysteine, Mass spectrometry, Liquid chromatography, Mouse tissues

## Abstract

S-Adenosylmethionine (SAM) serves as a methyl donor in biological transmethylation reactions. S-Adenosylhomocysteine (SAH) is the product as well as the inhibitor of transmethylations and the ratio SAM/SAH is regarded as the measure of methylating capacity (“methylation index”). We present a rapid and sensitive LC–MS/MS method for SAM and SAH determination in mice tissues. The method is based on chromatographic separation on a Hypercarb column (30 mm × 2.1 mm, 3 μm particle size) filled with porous graphitic carbon stationary phase. Sufficient retention of SAM and SAH on the chromatographic packing allows simple sample preparation protocol avoiding solid phase extraction step. No significant matrix effects were observed by analysing the tissue extracts on LC–MS/MS. The intra-assay precision was less than 9%, the inter-assay precision was less than 13% and the accuracy was in the range 98–105% for both compounds. Stability of both metabolites during sample preparation and storage of tissue samples was studied: the SAM/SAH ratio in liver samples dropped by 34% and 48% after incubation of the tissues at 4 °C for 5 min and at 25 °C for 2 min, respectively. Storage of liver tissues at −80 °C for 2 months resulted in decrease of SAM/SAH ratio by 40%. These results demonstrate that preanalytical steps are critical for obtaining valid data of SAM and SAH in tissues.

## 1 Introduction

S-Adenosylmethionine (SAM) and S-Adenosylhomocysteine (SAH) are metabolites involved in the conversion of methionine to homocysteine in the proximal part of the methionine cycle. S-Adenosylmethionine is synthesized from methionine and ATP in a reaction catalysed by methionine adenosyltransferase. SAM is providing methyl group moieties in several dozens of transmethylation reactions of crucial biological importance [1]. S-Adenosylhomocysteine, produced by the methyl group transfer, has been demonstrated to inhibit at various concentrations these reactions [1–4]. It has been proposed, that transmethylation reactions may be inhibited also by low ratio between SAM and SAH [1,5–7], sometimes regarded in literature as the “methylation index”. The SAH concentration and SAM/SAH ratio may be altered in various clinical conditions as a result of accumulation of homocysteine, which reacts with adenosine and forms SAH by the reverse action of the enzyme S-adenosylhomocysteine hydrolase.

During the past decade, animal models of several disorders of homocysteine metabolism became available for studying the pathogenesis of the respective enzyme deficiencies [8–11]. In contrast to human patients the animal models offer a unique opportunity to determine concentration of relevant metabolites including SAM and SAH also in tissues, which may help further elucidating the pathogenetic mechanisms in homocystinurias. The aim of this study was to develop an assay to monitor the levels of SAM and SAH in animal tissue samples and to explore the preanalytical conditions for this assay.

Various methods for the analysis of SAM and SAH have been published. These include LC methods with UV detection utilizing ion-pairing [12–17] or cation exchange chromatography [12,18], LC methods with fluorescence detection after conversion of the analytes to fluorescent analogs [5,19–21], LC methods with electrochemical detection [22] and capillary electrophoresis method [23]. More recently described sensitive LC–MS [24] and LC–MS/MS methods [25,26] enable quantification of SAM and SAH presented in plasma or cerebrospinal fluid in low nanomolar concentration, but require a SPE extraction step and sample volumes ranging from 250 μL to 1 mL. Because the polar SAM and SAH are just weakly retained on C_18_ columns without the use of ion-pairing additive in the mobile phase, SPE is primarily required in the LC–MS/MS applications to separate both analytes from salts and other early eluting matrix components that can lead to ion-suppression on mass spectrometer.

Other authors used penta-fluorinated column for LC–MS/MS analysis of SAM and SAH in mouse embryos [27]. Increased retention of SAM and SAH on this column, accomplished by the use of ion-pairing reagent – heptafluorobutyric acid – in the mobile phase, enables to simplify the sample preparation to tissue homogenization and heat precipitation of proteins. The limits of detection of the latter three LC–MS/MS methods are in the range of 2–10 and 1–2.5 nmol/L for SAM and SAH, respectively.

Our goal was to develop a sensitive LC–MS/MS method with minimal sample requirements and with sufficient sample throughput. Preferably, we tried to omit the SPE step, which demands usually higher sample and reagents volumes, prolongs labour time and increases the cost of the method. This was accomplished by the use of column filled with porous graphitic carbon (PGC) as a stationary phase, which retains both analytes and separates them from interfering compounds causing ion-suppression effects on mass spectrometer. In contrast to Burren et al. [27], no ion-pairing additives to the mobile phase were required to achieve sufficient retention of SAM and SAH on the column sorbent.

The methods for metabolite determinations in biological samples should be validated in detail focusing on stability of the metabolites in the sample matrix. A marked decrease of SAM and increase of SAH has been demonstrated in untreated plasma samples stored for 3 h at room temperature and for 1 month at −20 °C [26]. The acidification of the plasma with acetic acid to pH 4.5–5.0 stabilized both SAM and SAH in the samples for at least 4 months. In tissues, the metabolite changes may occur within seconds due to ischemic conditions during collection of tissue samples [15]. Rapid postmortem increase of SAM and SAH in mice tissues have been reported also by Helland and Ueland [28]. Therefore we tested the stability of SAM and SAH in excised tissues by incubating them at 4 and 25 °C for different time intervals.

If we have to use some cliché to describe the presented method, we would choose the attributes simple, cost effective and reproducible. This paper includes detailed sample preparation protocol with regard to demonstrated instability of SAM and SAH in tissue samples, and presents an original chromatographic separation of SAM and SAH utilizing simple mobile phases without ion-pairing reagents.

## 2 Material and methods

### 2.1 Chemicals

Acetonitrile (LC ultra-gradient grade) was purchased from J.T.Baker (Deventer, Netherlands). The internal standard [^2^H_3_]-SAM was purchased from CDN Isotopes (Pointe-Claire, Quebec, Canada), [^13^C_5_]-SAH was obtained from Dr. Herman J. ten Brink (VU Medical Center, Amsterdam, Netherlands). SAM *p*-toluenesulfonate salt for preparation of SAM standards as well as all other chemicals was obtained from Sigma–Aldrich (Prague, Czech Republic).

### 2.2 Apparatus and conditions

The LC–MS/MS system consisted of the Agilent 1100 Series LC System (Agilent Technologies, Palo Alto, CA, USA) coupled with API 3200 triple quadrupole mass spectrometer with electron ion source and operated with Analyst software, Vision 1.4 (Applied Biosystems, Foster City, CA, USA).

The separation was performed on a Hypercarb column (30 mm × 2.1 mm, 3 μm particle size, Thermo Fisher Scientific, Inc., USA) filled with porous graphitic carbon stationary phase. Gradient elution was composed of 0.1% formic acid in water (eluent A) and 0.1% formic acid in acetonitrile (eluent B). Gradient started with 100% A, followed by an increase to 32% B in 6 min. The column was than flushed with 80% B for 2 min and regenerated with 100% A for another 7 min. The flow rate was 0.3 mL/min. The eluent was diverted to waste at the beginning and end of each chromatographic run to prevent source contamination by salts and other compounds. Between the 5th and 10th min, the eluent was switched to the ion source of the mass spectrometer.

The detection of the analytes was carried out using positive electrospray ionization technique and selected reaction monitoring mode. The precursor → product transitions for SAM (*m*/*z* 399.3 → 250.3), SAH (*m*/*z* 385.3 → 136.3), [^2^H_3_]-SAM (*m*/z 402.3 → 250.3) and [^13^C_5_]-SAH (*m*/*z* 390.3 → 136.3) were monitored. The scan dwell time was set at 0.3 s for both the analytes and the internal standards. The optimized ion source parameters were: declustering potential: 42 and 40 V for SAM and SAH, respectively, collision energy: 25 and 27 V for SAM and SAH, respectively, entrance potential: 4.5 V, collision cell exit potential: 5.2 and 5.8 V for SAM and SAH, respectively, ionspray voltage: 4500 V and ionspray source temperature: 450 °C. The interface heater was set to 100 °C. The collision gas, curtain gas and ion source gas 1 and 2 (nebulizer gas and turbo gas) were set to 5, 14, 60 and 60 psi, respectively.

### 2.3 Standards and calibration curves

Stock solutions of SAM and SAH were prepared by dissolving approximately 4 mg of each compound in 1000 μl of ice cold water. The concentration of SAM and SAH in stock solution was determined by UV absorption spectroscopy at 260 nm using molar extinction coefficient of 15400 [29]. The calibration samples in the concentration range 1.25–320 μM were prepared by serial dilutions of the stock solution with 0.4 M perchloric acid (PCA), the individual calibration points were 1.25, 5, 10, 20, 40, 80, 160 and 320 μM. The calibration samples were aliquoted and stored at −80 °C. The calibration curve was obtained by weighted linear regression (weighing factor 1/*x*), the peak area ratio (analyte/internal standard) was plotted versus the analyte concentration.

### 2.4 Animals and tissue samples

Male C57BL/6J mice, aged 3–4 months, were used for determination of SAM and SAH in tissues and for experiments described in the section “Method validation”. Mice were maintained in a temperature- and light-controlled environment, they had free access to tap water and standard laboratory food. The experiments were approved by the Animal Care Committee of the 1st Faculty of Medicine. For the precision evaluation of the LC–MS/MS determination in samples with high SAM and SAH concentrations we used extracts from livers of cystathionine-ß-synthase deficient mice (C57BL/6J *Cbs*^−/−^) kindly provided by Dr. Jan P. Kraus, University of Colorado School of Medicine, USA.

### 2.5 Sample preparation

Mice were euthanized by decapitation, livers and kidneys were quickly excised and immediately immersed in liquid nitrogen. The snap frozen tissue samples were divided into pieces of approximately 50–150 mg. Frozen tissue aliquots were handled with utensils precooled in liquid nitrogen to prevent thawing of samples. Grinding of the frozen tissues to fine powder was performed in a precooled custom-made stainless steel pulverizer. Pulverized tissues were homogenized and deproteinized at once in ice-cold 0.4 M PCA (600 μL of PCA/100 mg of tissue). The homogenate was centrifuged 10 min at 7 000 × *g* at 10 °C and 30 μL of the supernatant was mixed with 10 μL of internal standard solution containing 50 μM of [^2^H_3_]-SAM and 75 μM of [^13^C_5_]-SAH. Before analysis, the sample was adjusted to pH 5–7 with 2.5 M K_3_PO_4_, left at 4 °C for 15 min to complete precipitation of potassium perchlorate and 5 μL of clear supernatant was injected on the LC column. If not analyzed immediately, the extracts were stored at −80 °C. The calibration samples were processed identically as the tissue sample extracts.

## 3 Results and discussion

### 3.1 Sample preparation

Protein precipitation is the simplest way of sample preparation for LC–MS/MS analysis. There are pros and cons which should be considered when choosing this approach instead of some of the other extraction methods. The pros are speed, reduction of sample and standard volumes to minimum resulting thus in cost effectiveness. The major disadvantage is the complexity of the sample matrix which may lead to ion suppression effects and to possible loss of sensitivity. We have chosen this option of sample preparation especially because of the minimal sample requirement. Using our sample preparation protocol, we obtained 300–900 μL of tissue extract. Only a small aliquot (30 μL) of the extract was spiked with labeled internal standards in order to minimize the consumption of the internal standard [^13^C_5_]-SAH which is expensive and commercially not readily available. The residual tissue extract can be used for determination of additional metabolites, as described previously [30].

Because of instability of SAM and SAH in tissues we performed all handlings with tissue specimens following the excision (including partitioning, weighing, grinding) in liquid nitrogen frozen samples. Tissue powderizing is required to prevent the formation of rubbery nugget in Potter-Elvehjem homogenizer. The homogenization was performed in denaturing ice-cold perchloric acid solution, in order to prevent enzymatic conversions of SAM and SAH during homogenization and further procedures. Moreover, acidic pH of perchloric acid stabilizes SAM, as previously reported [26].

### 3.2 Chromatography

**Fig. 1:**
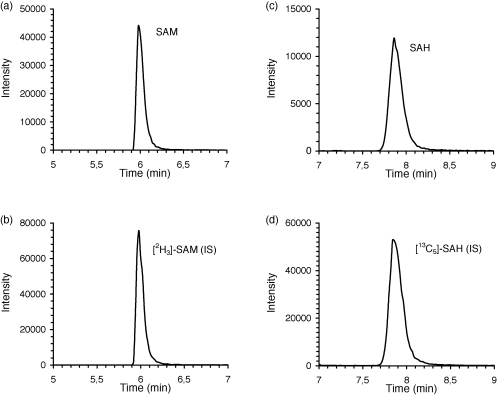
Chromatograms of SAM and SAH in mouse liver. The panels show selected reaction monitoring of the transitions *m/z* 399.3 → 250.3 for SAM (a); 402.3 → 250.3 for [^2^H_3_]-SAM (b); 385.3 → 136.3 for SAH (c) and 390.3 → 136.3 for [^13^C_5_]-SAH (d).

S-Adenosylmethionine is a polar compound and as such it is poorly retained on standard reversed-phase columns. High water content in the mobile phase and the presence of interfering substances close to the hold-up time (*t*_0_) result in poor ionization efficiency in the mass spectrometric detection. Some authors use ion-pairing additives in the mobile phase to improve the retention and also to increase the content of organic solvent in the mobile phase, however, these additives may cause loss of sensitivity on mass spectrometer [31]. We aimed at developing a simple LC method for separation of SAM and SAH without the use of ion-pairing reagents in the mobile phase. From the several tested columns we selected a column packed with porous graphitic carbon, which is a strong adsorbent offering retention mechanisms different from C_18_ stationary phases [32]. SAM was retained on the PGC column washed with 0.1% formic acid and was eluted by increasing the concentration of acetonitrile in the mobile phase. At 20% acetonitrile the retention of SAM dropped below 1 min, which we regarded as insufficient retention considering possible ionization suppression by compounds eluting at the *t*_0_. Because the retention factor of SAH was too high (more than 15 min) at acetonitrile concentrations lower than 20% in the mobile phase, we finally adopted a gradient elution to keep the retention of both metabolites in optimal range. After optimization of chromatographic conditions, the retention times of SAM and SAH were 5.9 and 7.8 min, respectively. The total chromatographic run time including column regeneration was 15 min. Typical chromatogram of mouse liver extract is shown in Fig. 1.

### 3.3 Method validation

#### 3.3.1 Linearity and limit of detection

The calibration curves were linear in the range 1.25–320 μM for both SAM and SAH. The calibration curve equation is *y* *=* *bx* *+* *c*, where *y* represents analyte/internal standard peak area ratio and *x* represents concentration of the analyte in μM. The mean equation (curve coefficients ± standard deviation) of the calibration curve (*n* = 5) was *y* = 0.0643(±0.0018)*x* + 0.0025(±0.0018) (correlation coefficient *r* = 0.999) for SAM and *y* = 0.0434(±0.0009)*x* − 0.0088(±0.0043) (correlation coefficient *r* = 0.999) for SAH.

The limit of detection (at a signal-to-noise ratio of 5:1) determined in standards diluted in 0.4 M PCA was 7.5 nM for SAM and 15 nM for SAH.

#### 3.3.2 Matrix-effects

Simplicity of the sample preparation protocol, which consists of protein deproteination and sample neutralization without the SPE step, brings in return the risk of increased matrix effects. We have evaluated the ion suppression effects by two methods.

Firstly, we compared the MRM signals in deproteinized tissue sample spiked with known SAM and SAH concentration (50 μM) to that of the analyte standards prepared in water. The comparison was done after subtracting the signals of endogenous SAM and SAH from the signals obtained in spiked sample. The results expressed as a mean(±standard deviation) of data obtained by replicate set of analyses (*n* = 5) showed enhancement of the SAM signal in spiked tissue extracts versus standards prepared in water by 9%(±4), whereas spiking of the tissue extracts with SAH resulted in small ionization suppression by 4%(±2). If analyte/I.S. ratios were used for the calculations as described above, the mean differences between data obtained from analysis of spiked tissue extracts and standard samples were lower than 2% for both analytes, showing that the matrix effects affecting the analyte signal were compensated by analogous enhancement or suppression of respective internal standards.

**Fig. 2:**
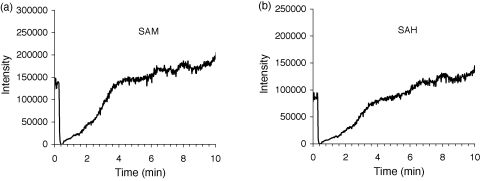
Infusion chromatograms showing the ion suppression effect. The column effluent was mixed with 200 μM standard solution of SAM and SAH, continuously introduced to the mass spectrometer interface. 5 μL of liver extract was injected onto the LC–MS/MS system. The drop in signal of the transition *m*/*z* 399.3 → 250.3 for SAM (a) and 385.3 → 136.3 for SAH (b) was monitored.

Secondly, we infused continuously a 100 μM standard solution of SAM and SAH by means of a syringe pump connected to the column effluent into the MS interface and injected the tissue sample into the LC system. The drop in the constant baseline signal would have indicated the ion suppression phenomenon [33]. This method gives information about the time intervals after sample injection, in which the ion suppression is manifested, and to what extent. Our results showed that the drop in signal in the first 2 min of analysis was more than 50%. At 4.5 min after sample injection, i.e. more than 1 min before the *t*_R_ of SAM and SAH, the signal practically returned to the initial level and remained unsuppressed until the end of analysis (Fig. 2).

#### 3.3.3 Precision and accuracy

We evaluated (a) the precision of the entire method, including sample preparation and LC–MS/MS determination and (b) the precision of the LC–MS/MS determination alone. The reason for this approach was to test whether the complex sample preparation protocol added significant imprecision to the LC–MS/MS determination alone. Because of ethical reasons, we performed the precision determination with limited number of liver samples excised from male C57BL/6J mice (see Section 2.4.). For the intra-day precision determination of the entire method we analyzed three pieces of liver tissue in one day and for inter-day precision we analyzed the fresh liver sample and aliquotes stored at −80 °C for 3 and 10 days.

To evaluate the precision of the LC–MS/MS method alone we used pooled tissue extracts with normal and high concentrations of SAM and SAH. The extract with normal SAM and SAH levels was obtained from liver of male C57BL/6J mice, the extract with high SAM and SAH levels was obtained from liver of cystathionine-ß-synthase deficient mice (see Section 2.4). The extracts were analyzed six times in the same analytical run (intra-day, LC–MS/MS) and on six separate runs within 6 weeks (inter-day, LC–MS/MS).

The results are given in Tables 1 and 2. The data show very good intra- and inter-day precision of the LC–MS/MS determination alone, the RSD did not exceed 5%. These results show very good stability of the tissue extracts stored at −80 °C for at least 6 weeks. Satisfactory performance below 9% was also obtained for the intra-day precision of the entire method, showing that the sample preparation protocol is reproducible and does not add a significant imprecision. The higher values of RSD calculated for inter-day precision for SAH determination (12.7%) may be explained by instability of SAH during storage of tissue samples at −80 °C for 10 days (see Section 3.3.4.2).

Accuracy was determined by spiking liver homogenate obtained from C57BL/6J mice with known concentrations of SAM and SAH. The concentrations of SAM and SAH in each spiked liver homogenate were determined by five replicate measurements. The determined concentration in the spiked samples was expressed as a percentage of the predicted concencentration, which was calculated as a sum of the added concentration and the endogenous level of the analyte in the unspiked sample. The results are given in Table 3. The calculated accuracy for both analytes was in the range of 98.4–104.9%.

#### 3.3.4 Sample stability

Sample stability studies were performed with tissues excised immediately from the sacrificed mice and incubated at either 25, 4 or −80 °C, respectively.

##### 3.3.4.1 Short-term stability of tissues at 25 and 4 °C

The time intervals, during which the tissues may have been exposed to non-freezing temperatures in the course of excision, weighing or homogenization, were not always specified in the previously published methodological studies. The stability study should demonstrate how the metabolite concentrations do change during these procedures and how meticulous the sample preparation protocol should be. In this study, liver and kidney tissues were incubated after excision from the mice at room temperature (25 °C) for 2 and 5 min and at 4 °C for 5 and 15 min, respectively.

The results are shown in Table 4. After incubation of liver tissues for just 2 min at 25 °C, there was a 17% decrease of SAM accompanied by a 60% increase in SAH concentration, resulting in a 48.1% decrease in the SAM/SAH ratio. These changes were even more evident after 5 min incubation, after which the SAM/SAH ratio decreased by 63.2%. Similar situation was observed during incubation at 4 °C – the SAM/SAH ratio dropped by 33.8% and 44.9% after incubation for 5 and 15 min, respectively. As shown in Table 4, analogous changes were observed also by incubating kidney samples at 25 and 4 °C. These results show high instability of SAM and especially of SAH in tissue samples at both 25 and 4 °C, demonstrating the need for specialized sample preparation protocol to prevent the metabolite conversions.

##### 3.3.4.2 Long-term stability of tissues stored at −80 °C

To evaluate the changes of SAM, SAH and SAM/SAH ratio during storage of tissues at −80 °C we analyzed tissue aliquots stored for 2 and 6 months, respectively. The results given in Table 4 show changes analogous to incubation of tissues at 25 and 4 °C: decrease of SAM, increase of SAH and resulting decrease of SAM/SAH ratio for 39.8 and 51.9% after 2 and 6 months, respectively.

### 3.4 Mice tissue concentrations of SAM and SAH.

The concentrations of SAM and SAH in mice tissues excised from male C57BL/6J mice, aged 3–4 months (see Section 2.4.) are shown in Table 5. The values are in agreement with data published in two papers [16,23], although other groups have found approximately twofold higher SAM levels in liver and kidney tissues compared to us [14,34]. Helland and Ueland [28] found 2 to 3 times lower concentrations of SAH in liver and kidney, i.e. 13 nmol/g wet weight and 1.9 nmol/g wet weight, respectively. These authors, however, used special protocol for isolation of “in situ” freezed tissues from anesthetized animals submerged in liquid nitrogen. Similarly, Delabar et al. reported lower SAH concentration in kidney, i.e. below 1 nmol/g wet weight, using “freeze-clamp-technique” for tissue collection [15]. This protocol prevents ischemia, which may result in increases of determined adenine nucleosides concentrations, including SAM and SAH. The latter two protocols of tissue isolation are not approved in our laboratory and could not have been adopted.

### 3.5 Method application

Presented method was used to determine tissue concentrations in mice tissues. The concentrations of SAM and SAH in tissue homogenates was in micromolar range whereas the limit of detection of our method is at least two magnitudes lower. The sensitivity of our method enables determination of SAM and SAH in whole blood extracts prepared by deproteinization of whole blood with equal volume of 0.6 M PCA, which was proved in pilot experiments. The whole blood concentrations of SAM and SAH are in the range of hundreds of micromoles, which is close to the detection limits of some published LC-UV methods, but still easily detectable using our LC–MS/MS method. The concentrations of SAH in plasma are, however several dozens of nanomoles and are too low to be determined using our method without further optimalization. Lowering the detection limit may be possible for example by the use of one of the variety of SPE methods, as described for example in previous publication [24].

## 4 Conclusions

The presented method allows precise and sensitive determination of SAM and SAH. Limits of detection of presented method −7.5 nM for SAM and 15 nM for SAH – are at least one order of magnitude lower than in previously described LC-UV methods used for determination of SAM and SAH and are comparable with the sensitivity of published LC–MS/MS methods.

The method comprises special sample preparation protocol to prevent metabolite changes due to demonstrated limited stability of the analytes in tissue samples. The stability studies show that tissues samples should be snap frozen in liquid nitrogen immediately after excision, kept frozen at −80 °C and processed by the described protocol as soon as possible, preferably within a week.

## Figures and Tables

**Table 1:** Intra-assay precision.

	*N*	nmol/g wet weight		RSD (%)	
		SAM	SAH	SAM	SAH
Entire method	4	60.6	27.2	8.8	7.8
LC–MS/MS^a^	6	38.9	65.4	3.9	2.2
LC–MS/MS^b^	6	251.4	998.5	4.5	1.2

The determinations for intra-assay precision studies were performed in liver tissue from C57BL/6J mouse (entire method) and liver extracts from C57BL/6J (LC–MS/MS^a^) and C57BL/6J *Cbs*^−/−^ (LC–MS/MS^b^) mice.

**Table 2:** Inter-assay precision.

	*N*	nmol/g wet weight		RSD (%)	
		SAM	SAH	SAM	SAH
Entire method	4	53.8	35.4	10.6	12.7
LC–MS/MS^a^	6	40.7	61.1	4	4.5
LC–MS/MS^b^	6	263.5	937.7	5.8	4.1

The determinations for inter-assay precision studies were performed in liver tissue from C57BL/6J mouse (entire method) and liver extracts from C57BL/6J (LC–MS/MS^a^) and C57BL/6J *Cbs*^−/−^ (LC–MS/MS^b^) mice.

**Table 3:** Accuracy data for the LC–MS/MS determination in liver extracts.

*N*	SAM		Accuracy (%)	SAH		Accuracy (%)
	Added (μM)	Mean ± SD measured (μM)		Added (μM)	Mean ± SD measured (μM)	
5	0	7.1 ± 0.2		0	10.4 ± 0.2	
5	10	16.9 ± 0.3	98.4	12.5	22.7 ± 0.4	98.9
5	80	91 ± 0.8	104.9	100	114.3 ± 5	103.9

The determinations for accuracy studies were performed in liver extract from C57BL/6J mouse spiked with known concentrations of SAM and SAH.

**Table 4:** Stability of SAM and SAH in mice tissues incubated at −80, 4 °C and 25 °C.

Liver						
	nmol/g wet weight			Difference (%)		
	SAM	SAH	SAM/SAH	SAM	SAH	SAM/SAH
Fresh liver	58.4	29.8	2.0			
2 min at 25 °C	48.5	47.7	1.0	−17.0	60.0	−48.1
5 min at 25 °C	46.6	64.7	0.7	−20.2	116.8	−63.2
Fresh liver	60.6	27.2	2.2			
5 min at 4 °C	64.2	43.2	1.5	6.0	59.1	−33.8
15 min at 4 °C	58.9	47.8	1.2	−2.8	75.9	−44.9

Fresh liver	60.3	40.7	1.5			
2 months at −80 °C	58.7	65.9	0.9	−2.6	61.8	−39.8
6 months at −80 °C	48.5	68.1	0.7	−19.5	67.3	−51.9

Kidney						
	nmol/g wet weight			Difference (%)		
	SAM	SAH	SAM/SAH	SAM	SAH	SAM/SAH
Fresh kidney	30.7	6.6	4.6			
2 min at 25 °C	31.9	8.6	3.7	4.1	30.0	−19.9
5 min at 25 °C	30.4	14.1	2.2	−0.9	113.2	−53.5
Fresh kidney	33.7	6.5	5.2			
5 min at 4 °C	29.2	7.8	3.7	−13.2	19.4	−27.7
15 min at 4 °C	29.6	8.9	3.3	−12.1	36.8	−36.1

**Table 5:** SAM and SAH levels in mice tissues (male C57BL/6J, aged 3–4 months).

	*N*	nmol/g wet weight	SD	nmol/g wet weight	SD
		SAM		SAH	
Liver	4	58.3	3.2	33.3	6.1
Kidney	4	33.7	3.1	6.9	0.5
